# Improved methods for estimating fraction of missing information in multiple imputation

**DOI:** 10.1080/25742558.2018.1551504

**Published:** 2018-11-23

**Authors:** Qiyuan Pan, Rong Wei

**Affiliations:** 1 National Center for Health Statistics (NCHS), 3311 Toledo Rd., Hyattsville, Maryland 20782, USA.; 2 NCHS, 3311 Toledo Rd., Hyattsville, MD 20782, USA.

**Keywords:** fraction of missing information, multiple imputation, missing data, National Ambulatory Medical Care Survey, number of imputations, Science, Mathematics & Statistics, Applied Mathematics, Mathematics for Biology & Medicine

## Abstract

Multiple imputation (MI) has become the most popular approach in handling missing data. Closely associated with MI, the fraction of missing information (FMI) is an important parameter for diagnosing the impact of missing data. Currently γ_*m*_, the sample value of FMI estimated from MI of a limited *m*, is used as the estimate of γ_0_, the population value of FMI, where *m* is the number of imputations of the MI. This FMI estimation method, however, has never been adequately justified and evaluated. In this paper, we quantitatively demonstrated that *E*(γ_*m*_) decreases with the increase of *m* so that *E*(γ_*m*_) > γ_0_ for any finite *m*. As a result *γ*_*m*_ would inevitably overestimate γ_0_. Three improved FMI estimation methods were proposed. The major conclusions were substantiated by the results of the MI trials using the data of the 2012 Physician Workflow Mail Survey of the National Ambulatory Medical Care Survey, USA.

## 1. Introduction

Multiple imputation (MI) becomes the most popular approach to accounting for missing data (Carpenter & Kenward, 2013, Dohoo, 2015, Rezvan, Lee, & Simpson, 2015, Rubin, 1987, Van Buuren, 2012). Closely associated with MI, fraction of missing information (FMI) is an important parameter for diagnosing the effects of data missingness (Rubin, 1987). FMI can be interpreted as the fraction of information about *Q* due to non-response, where *Q* is the quantity of interest (Rubin, 1987). As MI become increasingly important, the importance of FMI is also increasing. The best known use of FMI is to define the relative efficiency (RE) of MI as RE = (1 + *γ*_0_/*m*)^−1/2^, where *γ*_0_ is the population value of FMI and *m* is the number of imputations (Rubin, 1987). Based on this RE, Rubin concluded that *m* ≤ 5 would be sufficient for MI (Rubin, 1987). Little et al. as well as Wagner suggested that FMI be used as an alternative tool for measuring data missing data or the response rate (Little et al., 2016, Wagner, 2010). Siddique, Harel, Crespic, and Hedekerd (2014) used FMI to verify the missing data mechanisms. The most common practice of FMI estimation is to use ${\hat{\gamma }}_{0}=\gamma_{m}$, where ${\hat{\gamma }}_{0}$ is the estimated value of γ_0_ and γ_*m*_ is the FMI obtained from MI of a given *m*, e.g. (Khare, Little, Rubin, & Schafer, 1993, Lewis et al., 2014, Schafer, 2001, Schenker et al., 2006). However, the accuracy of the ${\hat{\gamma }}_{0}=\gamma_{m}$ method has not been adequately evaluated. This paper is to quantify possible biases of ${\hat{\gamma }}_{0}=\gamma_{m}$ and to improve FMI estimation methodology if necessary and possible.

Established by Rubin in 1987, the current FMI paradigm is defined by Equations (1)–(11) below:

(1) $${\bar{Q}}_{m}=\frac{1}{m}\sum_{m}^{1}Q_{i},$$

where subscript *m* and ∞ stands for a finite and infinite *m*, the subscript 0 for the population value, the subscript *i* for the *i*th imputation, and the bar hat for the parameter’s mean.

(2) $$B_{m}=\frac{1}{m-1}\sum_{1}^{m}(Q_{i}-{\bar{Q}}_{m}{)}^{2}$$

(3) $$U_{m}=\frac{1}{m}\sum_{1}^{m}U_{i}$$

(4) $$T_{m}=U_{m}+\left(1+\frac{1}{m}\right)B_{m},$$

where *B, U*, and *T* are the between-imputation, within-imputation, and the total variances.

(5) $$r=\left(1+\frac{1}{m}\right)\frac{B_{m}}{U_{m}},$$

where *r* is the fractional variance increase due to data missingness.

(6) $$v=(m-1){\left(1+\frac{1}{r}\right)}^{2},$$

where *v* is the degrees of freedom.

(7) $$\gamma_{m}=\frac{r+2/(v+3)}{r+1}$$

(8) $$T_{\infty }=U_{\infty }+B_{\infty }$$

(9) $$\gamma_{\infty }=\frac{B_{\infty }}{T_{\infty }}$$

(10) $$T_{0}=U_{0}+B_{0}$$

(11) $$\gamma_{0}=\frac{B_{0}}{T_{0}}.$$

Equation (11) cannot be used to calculate *γ*_0_ in practice because *B*_*0*_, *U*_*0*_, and *T*_*0*_ are usually unknown. No researchers have provided an equation that explicitly links *γ*_*m*_ and *γ*_0_. The justification for using ${\hat{\gamma }}_{0}=\gamma_{m}$ is not available from Equations (1)–(11).

Assume $\gamma_{\infty }=\gamma_{0}$. For ${\hat{\gamma }}_{0}=\gamma_{m}$ to be valid, *E*(*γ*_*m*_) = *γ*_0_ must be true. For *E*(*γ*_*m*_) = *γ*_0_ to be true, *E*(*γ*_*m*_) must be independent of *m*. To understand *E*(*γ*_*m*_), let the same MI of a given *m* be repeated for *j* times. Denote the *γ*_*m*_ from each MI repeat as *γ*_*m*1_, *γ*_*m*2_, *γ*_*m*3_, …, *γ*_*mj*_. By definition, the expected value is the sum of all possible values each multiplied by the probability of its occurrence (Hogg, McKean, & Craig, 2013). Therefore, *E*(*γ*_*m*_) can be defined as:

(12) $$E(\gamma_{m})=\underset{j\to \infty }{\lim}\left(\frac{1}{j}\sum_{j}^{1}\gamma_{mj}\right).$$

Equation (12) shows that *E*(*γ*_*m*_) can be understood as the ultimate mean of *γ*_*m*_ when *j* becomes infinity. If *E*(*γ*_*m*_) is independent of *m*, we should have *E*(*γ*_2_) = *E*(*γ*_3_) = … = *E*(*γ*_*m*_) = *γ*_0_, and the use of ${\hat{\gamma }}_{0}=\gamma_{m}$ would be justified. If *E*(*γ*_*m*_) depends on *m*, we should have *E*(*γ*_2_) ≠ *E*(*γ*_3_) ≠ … ≠ *E*(*γ*_*m*_) ≠ *γ*_0_. The use of ${\hat{\gamma }}_{0}=\gamma_{m}$ may not be justified if the difference between *E*(*γ*_*m*_) and *γ*_0_ is intolerably big.

Rubin indicated that the mean of *γ*_*m*_ can be regarded as *γ*_0_ [1 page 143], underlining an assumption that *E*(*γ*_*m*_) is independent of *m*. Harel briefly mentioned that *γ*_*m*_ “tends to decrease as *m* increases” without providing any details (Harel, 2007). Although Harel’s statement favours *E* (*γ*_*m*_) ≠ *γ*_0_, it cannot be a base for disproving ${\hat{\gamma }}_{0}=\gamma_{m}$ because it might be acceptable to use ${\hat{\gamma }}_{0}=\gamma_{m}$ if the decrease of *γ*_*m*_ with the increase of *m* is statistically negligible. To date the justifications for using ${\hat{\gamma }}_{0}=\gamma_{m}$ is still missing.

For FMI estimation, Harel’s 2007 paper (Harel, 2007) is important in that it pointed out that, unlike *γ*_*m*_ that “tends to decrease as *m* increases,” the quantity *B*_*m*_/(*U*_*m*_ + *B*_*m*_) “does not tend to decrease as *m* increases” (Harel, 2007). Harel used ${\hat{\gamma }}_{0}=B_{m}/(U_{m}+B_{m})$ to estimate FMI in his research (Harel, 2007). The goal of Harel’s paper was not to find a better FMI estimation method per se and his discussion on ${\hat{\gamma }}_{0}=B_{m}/(U_{m}+B_{m})$ was brief. Most researchers have not used Harel’s method for FMI estimation probably because most people may have treated Harel’s method as being research-specific rather than a method that may potentially be universally used for FMI estimation.

In this study, we examined the relationships between *m, γ*_*m*_, *γ*_∞_, and *γ*_0_, quantified the decrease of *γ*_*m*_ with the increase of *m*, quantified the biases of ${\hat{\gamma }}_{0}=\gamma_{m}$, and proposed improved methods for FMI estimation. Only univariate FMI definition will be examined in this paper even though multi-variate FMI definition may exist. The major conclusions were substantiated by the MI trials using the data of the 2012 Physician Workflow Mail Survey (PWS) of the National Ambulatory Medical Care Survey (NAMCS). This paper focuses on MI approach only even though it may be possible to estimate FMI via a non-MI approach (Savalei & Rhemtulla, 2012, Zheng & Lo, 2008).

## 2. The relationships between *m, γ*_*m*_, *γ*_∞_, and *γ*_0_

### 2.1. The condition for γ_∞_ = γ_0_

To use *γ*_*m*_ for estimating *γ*_0_, one must assume *γ*_∞_ = *γ*_0_. Most researchers simply treat *γ*_∞_ and *γ*_0_ as synonyms (e.g. He et al., 2016). But they are not. Imagine a population of imputations (POI) is generated by repeating the imputation of the same model on the same data for an infinite number of times. An MI is simply a sample of the POI with sample size *m*. The sample value and the population value of FMI for the POI are *γ*_*m*_ and *γ*_∞_, respectively. The population for *γ*_0_, however, is not POI but the population of the sampling units of the survey. A *γ*_∞_ is inseparably linked to an MI, but a *γ*_0_ can be independent of MI. The *γ*_0_ may be estimated by MI as well as other methods such as maximum likelihood (Savalei & Rhemtulla, 2012, Zheng & Lo, 2008). Using Equations (5)–(7), one can prove that the condition for *γ*_∞_ = *γ*_0_ is *B*_*m*_/*U*_*m*_ = *B*_0_/*U*_0_. When we use MI to estimate *γ*_0_, we have to assume *γ*_∞_ = *γ*_0_, which is probably why *γ*_∞_ and *γ*_0_ are often treated as synonyms in MI analyses. In this paper, we will assume *γ*_∞_ = *γ*_0_ because we use MI to estimate *γ*_0_.

### 2.2. B_m_/U_m_ is independent of m

Equation (7) that defines γ_*m*_ does not have *m* as a factor. Combining Equations (5), (6), and (7) gives an expanded definition of γ_*m*_ with *m* as one of the independent factors affecting γ_*m*_:

(13) $$\gamma_{m}=\frac{\left(1+\frac{1}{m}\right)\frac{B_{m}}{U_{m}}+2/\left((m-1){\left(1+\frac{1}{\left(1+\frac{1}{m}\right)\frac{B_{m}}{U_{m}}}\right)}^{2}+3\right)}{\left(1+\frac{1}{m}\right)\frac{B_{m}}{U_{m}}+1}.$$

Equation (13) shows that γ_*m*_ is a function of three factors, i.e. γ_*m*_ = *F*(*m, B*_*m*_, *U*_*m*_). In Equation (13), *B*_*m*_ and *U*_*m*_ always appear together as *B*_*m*_/*U*_*m*_. Letting *c*_*m*_ = *B*_*m*_/*U*_*m*_, then γ_*m*_ becomes a function of two factors, i.e. γ_*m*_ = *F*(*m, c*_*m*_).

Whether *c*_*m*_ is independent of *m* is important in understanding the *m*–γ_*m*_ relationship. If *c*_*m*_ depends on *m*, the direct effects of *m* on γ_*m*_ would be confounded by the indirect effects of *m* on γ_*m*_ via *m*’s effects on *c*_*m*_, which in turn could be due to *m*’s effect on *B*_*m*_, *U*_*m*_ or both. If *c*_*m*_ is independent of *m*, then the *m*–γ_*m*_ relationship would be greatly simplified.

In order to establish that *c*_*m*_ is independent of *m*, we need to prove *E*(*B*_*m*_/*U*_*m*_) = *B*_0_/*U*_0_. Equation (2) indicates that the relationship between *m* and *B*_*m*_ is that between the sample size *n* and the variance (*s*^2^) so that *E*(*B*_*m*_) is independent of *m*, i.e. *E*(*B*_*m*_) = *B*_0_ (Serfling, 1980). Equation (3) indicates that the relationship between *m* and *U*_*m*_ is that between the sample size *n* and the sample mean $\bar{x}$ so that *E*(*U*_*m*_) is independent of *m*, i.e. *E*(*U*_*m*_) = *U*_0_ (Hogg et al., 2013). Jensen’s Inequality (Hogg et al., 2013) determines that *E*(1/*U*_*m*_) ≥ 1/*E*(*U*_*m*_). Therefore *E*(*B*_*m*_/*U*_*m*_) = *E*(*B*_*m*_)*E*(1/ *U*_*m*_) ≥ *E*(*B*_*m*_)/*E*(*U*_*m*_) = *B*_0_/*U*_0_, or *E*(*B*_*m*_/*U*_*m*_) ≥ *B*_*0*_/*U*_*0*_. Our simulation studies show that the maximum difference between *E*(*B*_*m*_)/*E*(*U*_*m*_) and *E*(*B*_*m*_/*U*_*m*_) is less than 0.1%, which is negligible in virtually any statistics work. We can safely regard *E*(*B*_*m*_/*U*_*m*_) = *B*_0_/*U*_0_ as a fact for the purpose of studying the *m*– γ_*m*_ relationship. The *c*_*m*_’s independence of *m* is thus proved. The subscript *m* can be removed from *c*_*m*_. As a result, we can indeed letting *c*_*m*_ = *B*_*m*_/*U*_*m*_ be a constant *c* in Equation (13) and make γ_*m*_ become a function of the single factor *m*, i.e. γ_*m*_ = *F*(*m*).

### 2.3. The γ_m_ = F(m,γ_0_) equation

When *m* goes infinite, γ_*m*_ becomes γ_0_. Our goal is to establish the mathematic relationship between γ_*m*_ and γ_0_ at a finite *m*, which is currently missing in published literatures. In the discussions above, we have showed that it is mathematically legitimate to letting *c*_*m*_ be a constant *c* in studying the *m*– γ_*m*_ relationship because *c*_*m*_ is independent of *m*. What is the best value to choose for *c* to obtain the most truthful *m*–γ_*m*_ relationship? The answer is: *c* = *E*(*B*_*m*_/*U*_*m*_) = *B*_0_/*U*_0_. If and only if *c* = *E*(*B*_*m*_/*U*_*m*_) = *B*_0_/ *U*_0_, the *m*–γ_*m*_ relationship as determined by Equation (13) would reflect the true *m*–γ_*m*_ relationship. From Equations (10) and (11) we can obtain *B*_0_/*U*_0_ = γ_0_/(1 − γ_0_). Replacing *B*_*m*_/*U*_*m*_ in Equation (13) with γ_0_/(1 − γ_0_), we obtain an equation that directly links γ_*m*_ to γ_0_ as follows:

(14) $$\gamma_{m}=E(\gamma_{m})=F(m,\gamma_{0})=\frac{\left(1+\frac{1}{m}\right)\frac{\gamma_{0}}{1-\gamma_{0}}+2/\left((m-1){\left(1+\frac{1}{\left(1+\frac{1}{m}\right)\frac{\gamma_{0}}{1-\gamma_{0}}}\right)}^{2}+3\right)}{\left(1+\frac{1}{m}\right)\frac{\gamma_{0}}{1-\gamma_{0}}+1}.$$

Establishment of equation is a significant step forward in understanding the relationship between *m,* γ_*m*_, and γ_0_ because it links the three factors in the same equation for any *m*, finite or infinite.

For a given analysis of a given dataset, γ_0_ is a constant. When we repeat the same MI of a given *m* for *j* times, the γ_*m*_ value from each repeat of the MI will not change when the γ_*m*_ is determined by Equation (14). In other words, we will have γ_*m*1_ = γ_*m*2_ = γ_*m*3_ = … γ_*mj*_ = *E*(γ_*m*_) (see Equation (12)). In other words, the γ_*m*_ value obtained from Equation (14) will be *E*(γ_*m*_). For different data and analyses, γ_0_ is a variable. Equation (14) shows that *E*(γ_*m*_) is a function of the two factors, *m* and γ_0_, i.e. *E*(γ_*m*_) = *F*(*m,* γ_0_).

## 3. The decrease of *E*(γ_*m*_) with the increase of *m*

### 3.1. E(γ_m_) > γ_0_ for any finite m

**Figure 1.**
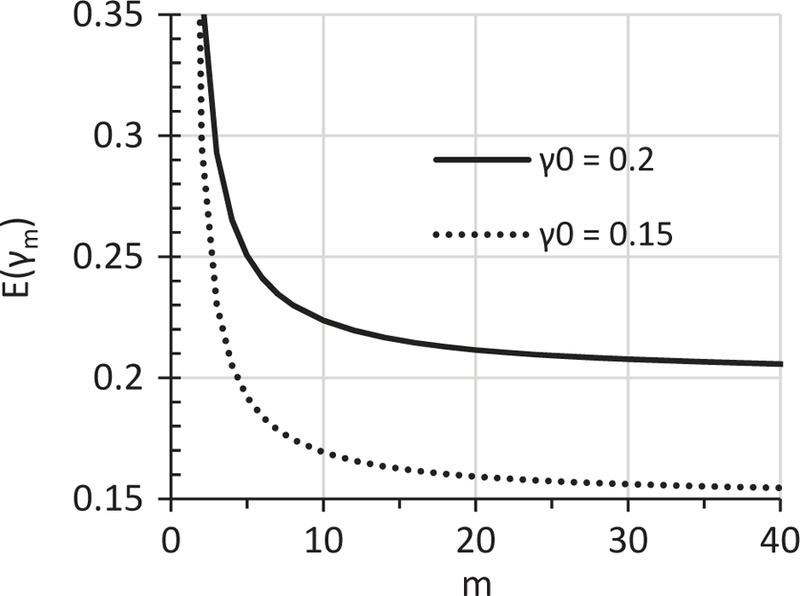
The *m*–*E*(γ_*m*_) relationship curve at γ_0_ = 0.2 and 0.15 as determined by Equation (14).

We all know that $E(\bar{x})$ is independent of *n* and equals to μ, which provides the theoretical base for $\hat{\mu }=\bar{x}$ (Hogg et al., 2013). The use of ${\hat{\gamma }}_{0}=\gamma_{m}$ implies the assumption that *E*(γ_*m*_) = γ_0_. Using Equation (14), the *m*–*E*(γ_*m*_) relationship curve can be constructed for any given γ_0_. Figure 1 presents the *m*–*E*(γ_*m*_) relationship curves for γ_0_ = 0.15 and 0.2. Based on Figure 1, for the first time in MI research, we can explicitly state this important fact: *E*(γ_*m*_) decreases with the increase of *m*. The decrease of *E*(γ_*m*_) with the increase of *m* can be interpreted as follows: For a given dataset with a given MI model, the ultimate mean of γ_*m*_, which is the mean of an infinite number of individual γ_*m*_ values obtained from repeating the MI of the given *m* for an infinite number of times, would always be greater than the γ_0_. Of course what is called “the ultimate mean” here is the *E*(γ_*m*_) (Hogg et al., 2013). Therefore, by showing *E*(γ_*m*_) decreases with the increase of *m*, we have proved that *E*(γ_*m*_) > γ_0_ for any finite *m* (Figure 1). The fact that *E*(γ_*m*_) > γ_0_ is further illustrated by more data in Table 1 for a wider range of *m* values and more γ_0_ values.

### 3.2. The bias of the current FMI estimation method

The fact that *E*(γ_*m*_) > γ_0_ dictates that the current FMI estimation method ${\hat{\gamma }}_{0}=\gamma_{m}$ must be biased. One achievement of this paper is that we successfully quantified the bias of the current FMI estimation method. We use *D*_γ_, the percentage difference between *E*(γ_*m*_) and γ_0_ as the parameter to measure this bias, i.e.:

(15) $$D_{\gamma }=100\frac{\gamma_{m}-\gamma_{0}}{\gamma_{0}}.$$

**Figure 2.**
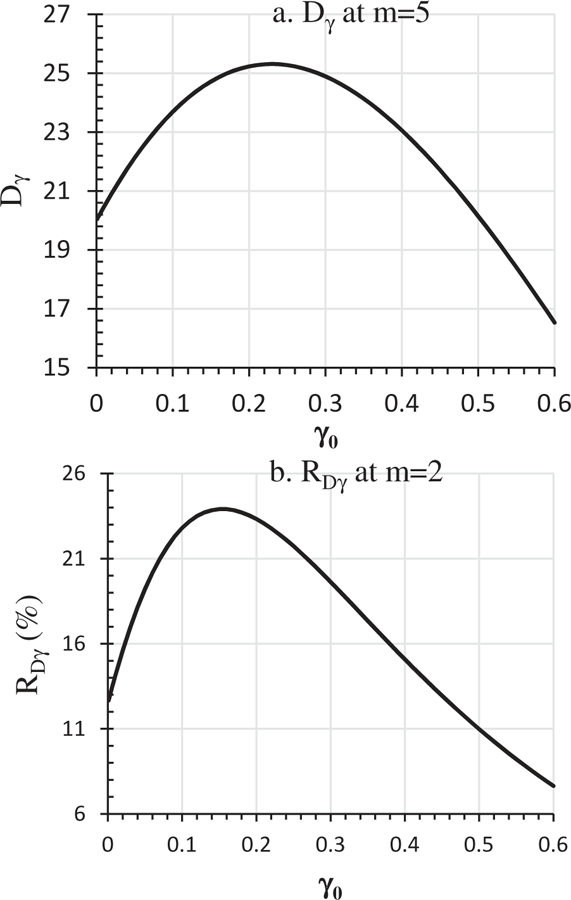
Effects of γ_0_ levels on*D*_γ_ as defined by Equation (15) and *R*_Dγ_ as defined by Equation (16): a. *D*_γ_ at *m* = 5; b. *D*_γ_ at *m* = 2.

Table 1 presents the *D*_γ_ values at different γ_0_ and *m* values as determined by Equation (14). At a given *m, D*_γ_ differs at different γ_0_ values (Table 1). For *m* = 2, *D*_γ_ is 80.59% and 53.64% for γ_0_ = 0.2 and 0.01, respectively (Table 1). When γ_0_ increased from 0.001 to 0.6, *D*_γ_ first increases with the increase of γ_0_, reaches a peak, and then decreases (Table 1 and Figure 2). The value of the γ_0_ at which *D*_γ_ reaches the peak differs with *m* (data not shown). For *m* = 5, the maximum *D*_γ_ value of 25.31% occurs at γ_0_ = 0.23, and the minimum *D*_γ_ value of 16.53% occurs at γ_0_ = 0.6 (Figure 2(b)). In other words, one could overestimate FMI by 25% at *m* = 5 if the current method is used. A bias of this magnitude cannot and should not be ignored. Development of a better FMI estimation method is indeed necessary.

### 3.3. The γ_m_ decrease rate: smaller at larger m

We use *R*_γ*d*_, the percentage rate of the γ_*m*_ decrease per unit *m*, to measure the rate of the γ_*m*_ decrease:

(16) $$R_{D\gamma }=100\frac{\gamma_{m}-\gamma_{m+1}}{\gamma_{m+1}}.$$

$R_{D\gamma }$ is affected by both *m* and γ_0_ (Table 1 and Figure 2, b1 and b2). At *m* = 5, $R_{D\gamma }$ is 3.87% and 2.96% for γ_0_ = 0.2 and 0.01, respectively (Table 1). Figure 2(b) show that $R_{D\gamma }$ increases initially, reaches a peak, and then decreases as γ_0_ increases from 0.001 to 0.6. For *m* = 2, the maximum $R_{D\gamma }$ = 23.91% occurs at γ_0_ = 0.15 (Figure 2(b)). For *m* = 5, the maximum $R_{D\gamma }$ = 3.88% occurs at γ_0_ = 0.21 (data not shown). The gradual reduction of $R_{D\gamma }$ makes it possible for choosing a sufficient *m* when the *m*-driven γ_*m*_ reduction becomes negligibly small.

## 4. Improved methods for γ_0_ estimation

Regarding ${\hat{\gamma }}_{0}=\gamma_{m}$ as the control, any method that gives more accurate FMI estimation than this control will be considered as an improved method. Three improved methods are proposed below.

### 4.1. Improved method 1: ${\hat{\gamma }}_{0}=\gamma_{m\geq 100}$

The control method is to use ${\hat{\gamma }}_{0}=\gamma_{m}$ regardless the size of *m*. The first improved method is to choose a sufficiently large *m* when use ${\hat{\gamma }}_{0}=\gamma_{m}$. Data in Figure 1 show that *E*(γ_*m*_) approaches γ_0_ as *m* gets larger. Therefore, γ_*m*_ would estimate γ_0_ with an adequate accuracy when *m* is sufficiently large. Various criteria have been used to determine the sufficient *m* (Bodner, 2008, Graham, Olchowski, & Gilreath, 2007, Hershberger & Fisher, 2003, Pan, Wei, Shimizu, & Jamoom, 2014, Royston, 2004, Rubin, 1987). An adequately accurate estimation of γ_0_ using ${\hat{\gamma }}_{0}=\gamma_{m}$ offers another criterion for determining a sufficient *m*. As measured by $R_{D\gamma }$, the gain in reducing the bias from increasing a unit *m* becomes smaller at a greater *m*. Using Equation (14), we can prove the bias of the default method as measured by *D*_γ_ would be about 1% or less for any reasonable γ_0_ values when *m* is greater than 100. We arbitrarily choose a bias of ≤1% as an acceptable level and recommend *m* ≥100 as being sufficient for an adequately accurate estimation of γ_0_ using ${\hat{\gamma }}_{0}=\gamma_{m}$. This method can be expressed as ${\hat{\gamma }}_{0}=\gamma_{m\geq 100}$.

### 4.2. Improved method 2: ${\hat{\gamma }}_{0}=\gamma_{m}\left(m/\left(m\;+1\right)\right)$

Calculating γ_*m*_ for different *m* and γ_0_ combinations using Equation (14), one will find the following approximation stands well for *m* ≥10:

(17) $$\gamma_{m}\approx \frac{m+1}{m}\gamma_{0}.$$

From Equation (17), we obtain the following method of estimating γ_0_ from γ_*m*_:

(18) $${\hat{\gamma }}_{0}=\frac{m}{m+1}\gamma_{m}.$$

For those who may be interested, this method may be proven by resolving γ_0_ from Equation (14) using Taylor series expansion approximation. An advantage of this method is that one could use it to have a more accurate FMI estimation from the *m* and the γ_*m*_ information available in an earlier publication that uses a small *m* and γ_*m*_ to estimate FMI.

### 4.3. Improved method 3: $\hat{\gamma }=c_{m}/(c_{m}+1)$, where c_m_ = B_m_/U_m_

In Section 2.2, we proved that *E*(*B*_*m*_/*U*_*m*_) = *B*_*0*_/*U*_*0*_. In other words, *B*_*m*_/*U*_*m*_ is an unbiased estimation of *B*_*0*_/*U*_*0*_. As a result, Equation (19) below is a better γ_0_ estimation than ${\hat{\gamma }}_{0}=\gamma_{m}$:

(19) $${\hat{\gamma }}_{0}=\frac{c_{m}}{1+c_{m}}.$$

where *c*_*m*_ = *B*_*m*_/*U*_*m*_. Harel used this method to estimate γ_0_ for his study on two-stage MI (Harel, 2007). However, the justification for this method discussed here was not available in Harel’s paper or any other published literature (Harel, 2007).

## 5. Results from MI trials of PWS12

### 5.1. Methods

PWS was a supplemental survey of NAMCS, which collects data about the provision and use of ambulatory medical care services in the United States (Lau, McCaig, & Hing, 2016). The 2012 PWS data (PWS12) were used for the MI trial, which had 2,567 responded physicians in the sample. PWS data can be accessed via NCHS Research Data Center (RDS) program (https://www.cdc.gov/rdc/index.htm).

MI was conducted on three variables representing the physician’s practice size at different scales, namely SIZE100, SIZE20, and SIZE5. The three variables had the same missing data percentage of 29% due to item non-responses. The hot-deck imputation method (Andridge & Little, 2010) was used. The RDS-released PWS12 data, which had 3.6% of missing values for SIZE after some of the missing values in PWS12 were replaced by the corresponding non-missing values for the same physician from the 2011 PWS data, were used as the hot-deck donor. Two MI models denoted as MI-1 and MI-2 were used. MI-1 did not use any covariate in the imputation and the non-missing replacement values for the missing value were randomly chosen from entire donor dataset. MI-2 used PRIMEMM as the covariate in the imputation and the non-missing replacement values for the missing value were randomly chosen from the cell of the same PRIMEMM value in donor dataset. PRIMEMM was the physician’s primary employment type that was coded into nine categories for this research. The MIs had *m* = 3, 5, 10, 20, 30, 40, 60, 80, and 100, with each MI being repeated for 30 times. Excluding *m*, there were 12 treatment combinations (3 imputed variables × 2 imputation models × 2 analytic models). The hot-deck imputation method used in this study was similar to that used by the survey for creating the RDC-released PWS12 data. According to Rubin (1987, equation 4.3.8), the hot-deck bias can be expressed as *E*(*B*)= *B*(*n*1/*n*), where *n* is the number of the units of the full sample and *n*1 is the number of the units with observed values. Since the *n*1/*n* ratio is independent of *m*, the percentage fraction of the hot-deck bias would be a fixed value as long as the *n*1/ *n* ratio is fixed. Therefore the *m*–γ_*m*_ relationship obtained from the hot-deck-based MI trials should still be valid. One should be aware of the potential hot-deck bias when interpreting the results of this study.

The quantity of interest (*Q*) was the means of the SIZE100, SIZE20, and SIZE5. Two analytical models denoted as Anal-1 and Anal-2 were used. In Anal-1, *U*_*i*_, the within-imputation variance of the *i*th complete dataset generated by the MI, was the total variance of SIZE100, SIZE20, or SIZE5 in the *i*th dataset. In Anal-2, *U*_*i*_ was the variance of the *i*th dataset after the variance due to the effect of PRIMEMM was removed. Analyses were based on un-weighted data. Results obtained in this study were for research purpose only.

Barnard and Rubin (1999) suggested that, for making the statistical inferences in MI-involved analyses, instead of using the degrees of freedom (*v*) as defined by Equation (6), the adjusted degrees of freedom (DFa) as proposed by their paper should be used where the complete-data degrees of freedom is not sufficiently large. However in the γ_*m*_ definition, i.e. Equation (7), *v* does not function as the degrees of freedom per se but mealy as a mathematical value in the estimation of γ_*m*_. We have found that replacing *v* in Equation (7) with DFa will result in an erroneous estimation of γ_*m*_. Therefore we used *v*, instead of DFa, when used Equation (7) for the γ_*m*_ estimation in this study.

### 5.2. The γ_m_ decrease with the increase of m in the MI trials

**Figure 3.**
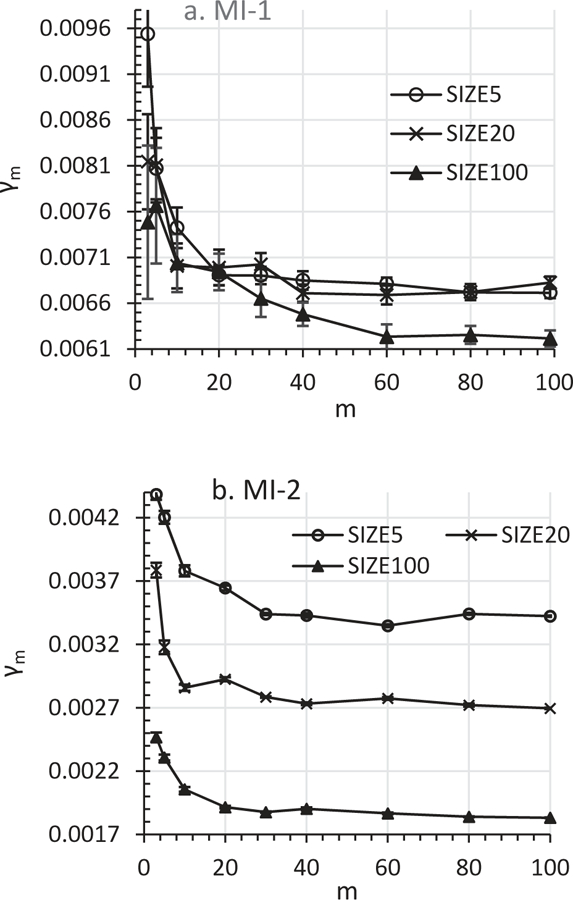
Effects of *m* on γ_*m*_ at δ = 29% for analytic model = Anal-2: MI model = MI-1; b. MI model = MI-2.

Would the γ_*m*_ decrease due to the increase of *m* (see Section 2.1 and 3.1) be big enough to stand out from sampling errors and other noises in real-world MI analyses? The answer is yes, as demonstrated by the data in Figure 3. Figure 3 shows the effects of *m* on γ_*m*_ in SIZE100, SIZE20, and SIZE5 for the two MI models for Anal-2. In spite of the γ_*m*_ variations due to sampling errors as shown by the error bars in the graphs, the dominant trend was clear: γ_*m*_ decreased significantly as *m* increased from 3 to 100. The γ_*m*_ values at *m* = 3–40 were significantly greater than γ_100_ in most cases (Figure 3). These results suggest that the γ_*m*_ decrease with the increase of *m* is not ignorable in FMI estimation in real world data analyses.

### 5.3. Variation of γ_m_, B_m_, and U_m_

In establishing the MI framework, Rubin (1987) assumed that *U*_*m*_ ≈ U_0_, which would be more likely to be true if the variance of *U*_*m*_ is negligible. The authors did not find any information on the magnitude of *U*_*m*_ variance in published literature. A detailed study on *B*_*m*_ variance was reported by Pan et al. (Pan et al., 2014). The variance of *B*_*m*_ was substantial when *m* < 30 (Pan et al., 2014). The variations in *B*_*m*_ and *U*_*m*_ would inevitably lead to γ_*m*_ variation. As a result, when using ${\hat{\gamma }}_{0}=\gamma_{m}$ at an insufficient *m*, the inaccuracy of ${\hat{\gamma }}_{0}$ would not only come from *E*(γ_*m*_) > γ_0_ but also from the variation of γ_*m*_ The possible bias from sampling-error-driven γ_*m*_ variation has not been given an adequate attention.

The coefficient of variations (CV) of *B*_*m*_, *U*_*m*_, and γ_*m*_ are presented in Table 2. Both the imputations models and the analytic models affected the variations of γ_*m*_, *B*_*m*_, and *U*_*m*_ (Table 2). CV of *U*_*m*_ was much smaller—usually 1–10% that of *B*_*m*_. The CV of *B*_*m*_ and γ_*m*_ were very similar, with the CV of γ_*m*_ being always slightly smaller than that of *B*_*m*_. The greater the *m*, the smaller the variations of γ_*m*_, *B*_*m*_, and *U*_*m*_ (Table 2). These results were in agreement with Harel’s conclusion (Hogg et al., 2013) that it is necessary to choose a sufficient *m* for MI to control the variations of γ_*m*_. Due to the significant effects of the MI model and the analytic model on the variations of γ_*m*_, *B*_*m*_, and *U*_*m*_ (Table 2), it may not be possible to propose a single *m* that fits all situations for controlling the variance of γ_*m*_, *B*_*m*_, and *U*_*m*_.

An advantage of using ${\hat{\gamma }}_{0}=\gamma_{m\geq 100}$ is that not only can this method reduce the *E*(γ_*m*_) > γ_0_ bias but also reduce γ_*m*_ variation because of a large *m*. The other two improved methods can effectively reduce or even eliminate the *E*(γ_*m*_) > γ_0_ bias even if when *m* is small. However the ${\hat{\gamma }}_{0}$ inaccuracy may be a concern for any FMI estimation methods unless a sufficient *m* is chosen. Data in Figure 3 suggest that a ≥20 *m* may be necessary to reduce the γ_*m*_ variation to an acceptable level for using ${\hat{\gamma }}_{0}=\gamma_{m}\left(m/\left(m+1\right)\right)$ and ${\hat{\gamma }}_{0}=c_{m}/\left(c_{m}+1\right)$.

### 5.4. Comparison of different FMI estimation methods

Table 3 presents data for visualizing the performance of these three improved γ_0_ estimation methods described in Section 4 in comparison with the default method ${\hat{\gamma }}_{0}=\gamma_{m}$ in an example of real-world data analyses. The treatment combination of the MI trials was {SIZE20, MI-2, Anal-2}. The control values was the γ_*m*_ values at *m* = 3, 5, etc., which would be the FMI estimation when the default method was used. The γ_100_ value was used as the ${\hat{\gamma }}_{0}$ for the improved method ${\hat{\gamma }}_{0}={\gamma_{m}}_{\geq 100}$. The best ${\hat{\gamma }}_{0}$ was calculated by Equation (19) using $\left({\bar{B}}_{100})/({\bar{U}}_{100}\right)$ as the estimate of *B*_0_/*U*_0_, where ${\bar{B}}_{100}$ and ${\bar{U}}_{100}$ were the mean of the 30 replicates of *B*_100_ and *U*_100_.

For *m* ≤ 80, all three improved methods performed better than the control method (Table 3). These results suggest that the three improved methods proposed in this paper can be used to replace the control method in real world data analyses. In general we recommend to use ${\hat{\gamma }}_{0}=c_{m}/\left(1+c_{m}\right)$, for it essentially eliminates the *E*(γ_*m*_) > γ_0_ bias at all levels of *m*. But the two other methods may come in handy under certain circumstances. For example, if an earlier publication which had used *m* = 5 without providing *B*_*m*_ and *U*_*m*_ values, one can simply use ${\hat{\gamma }}_{0}=\gamma_{m}m/\left(1+m\right)$ to convert the biased γ_0_ estimate of the paper into a more correct γ_0_ estimate.

## 6. Conclusions

In most published researches, γ_∞_ and γ_0_ are treated as synonyms. However, the two are different. The γ_0_ is independent of MI, whereas γ_∞_ is a parameter of MI. γ_∞_ equals to γ_0_ only if *B*_*m*_/*U*_*m*_ = *B*_0_/*U*_0_. To use MI for FMI estimation, one has to assume γ_∞_ = γ_0_, which will also be the assumption here.

The γ_*m*_ decreases with the increase of *m*. We quantified the *m*–γ_*m*_ relationship. The magnitude and the rate of the γ_*m*_ decrease varies with *m* and γ_0_. At *m* = 2, γ_2_ is greater than γ_0_ by 50–81% depending on the γ_0_ level. At *m* = 5, the recommended *m* value as being sufficient by some (e.g. Rubin, 1987), γ_*m*_ is greater than γ_0_ by 20–25% when γ_0_ value ranges from 0.001 to 0.6. The decrease of γ_*m*_ with the increase of *m* determines that *E*(γ_*m*_) > γ_0_ for any finite *m*. The results from the MI trials suggest that the volume of the γ_*m*_ decrease with increased *m* is not ignorable in real world data analyses in spite of the noises from sampling errors and other sources.

*E*(*B*_*m*_) and *E*(*U*_*m*_) are independent of *m*. Therefore, the decrease of γ_*m*_ with the increase of *m* is not due to an indirect effect of *m* on *E*(*B*_*m*_) and *E*(*U*_*m*_). As a result, it is not necessary to use the *B*_*m*_ and *U*_*m*_ from the same MI for best γ_*m*_ estimation. Instead, one should use the best estimates of *B*_0_ and *U*_0_ available, which leads to the development of Equation (14) that links γ_*m*_ to γ_0_ directly.

The variation in γ_*m*_ can be substantial. The CV of γ_*m*_ was essentially identical with that of *B*_*m*_, and CV of *U*_*m*_ was 1–10% that of γ_*m*_ or *B*_*m*_. The variation of γ_*m*_ is smaller as *m* gets bigger. The inaccuracy of FMI estimation due to γ_*m*_ variation should be concerned in FMI estimation when *m* is small regardless what method is used.

The current method ${\hat{\gamma }}_{0}=\gamma_{m}$ may result in a substantial FMI overestimation when *m* is not sufficiently large. Three improved methods are proposed for estimating γ_0_ from MI of a finite *m*. These three methods are (1)${\hat{\gamma }}_{0}=\gamma_{m\geq 100}$, (2)${\hat{\gamma }}_{0}=\gamma_{m}\left(m/\left(m+1\right)\right)$, and (3)${\hat{\gamma }}_{0}=c_{m}/\left(c_{m}+1\right)$, where *c*_*m*_ = *B*_*m*_/*U*_*m*_. In our MI trials, all three improved methods gave more accurate γ_0_ estimates than ${\hat{\gamma }}_{0}=\gamma_{m}$ where *m* is less than 80.

When *m* is sufficiently large, say, *m* ≥ 100, all three methods should give a statistically sound estimation of γ_0_. When *m* is not sufficiently large, say, *m* < 100, the third method ${\hat{\gamma }}_{0}=c_{m}/\left(c_{m}+1\right)$ should be one’s best option for γ_0_ estimation. The second method ${\hat{\gamma }}_{0}=\gamma_{m}\left(m/\left(m+1\right)\right)$ has its value where *B*_*m*_ and *U*_*m*_ are not available and the only values available to use for γ_0_ estimation are *m* and γ_*m*_.

## Figures and Tables

**Table 1.** Changes of *E*(γ_*m*_), *D*_γ_, and *R*_*D*γ_ with the increase of *m* at different γ_0_ levels, where *D*_γ_ = 100(*E*(γ_*m*_)−γ_0_ )/γ_0_ and *R*_*Dγ*_= 100(γ_*m*_ −γ_*m+1*_)/γ_*m*+1_

*m*	*E*(γ_*m*_)		*R*_*D*γ_		*D*_γ_	
	γ_0_ = 0.2	γ_0_ = 0.01	γ_0_ = 0.2	γ_0_ = 0.01	γ_0_ = 0.2	γ_0_ = 0.01
**2**	0.361	0.01536	23.33	14.12	80.59	53.64
**5**	0.250	0.01205	3.872	2.957	25.23	20.47
**10**	0.224	0.01102	1.0240	0.8502	11.84	10.16
**20**	0.212	0.01051	0.2664	0.2306	5.750	5.062
**40**	0.206	0.01025	0.0682	0.0602	2.837	2.528
**60**	0.204	0.01017	0.0306	0.0272	1.883	1.684
**100**	0.202	0.01010	0.0111	0.0099	1.126	1.010
**200**	0.201	0.01005	0.0028	0.0025	0.561	0.505

**Table 2.** Coefficient of variations (%) of *B*_*m*_, *U*_*m*_, and γ_*m*_ for SIZE100

*m*	MI-1, Anal-1			MI-2, Anal-2		
	*B*_*m*_	*U*_*m*_	γ_*m*_	*B*_*m*_	*U*_*m*_	γ_*m*_
3	20.24	0.185	20.19	1.52	0.0553	1.50
5	11.12	0.179	11.10	1.00	0.0200	1.01
10	7.75	0.135	7.75	0.88	0.0353	0.91
20	5.91	0.098	5.89	0.49	0.0282	0.48
30	6.24	0.077	6.26	0.24	0.0150	0.24
40	3.60	0.048	3.59	0.61	0.0180	0.59
60	2.74	0.058	2.73	0.39	0.0091	0.39
80	2.48	0.041	2.47	0.17	0.0062	0.17
100	2.45	0.037	2.45	0.15	0.0081	0.16

**Table 3.** Comparison of different γ_0_ estimation methods for SIZE20 with imputation model = MI-2 and analytic model = Anal-2 in the PWS12 MI trials. The best ${\hat{\gamma }}_{0}$ was calculated by Equation (19) using $\left({\bar{B}}_{100})/({\bar{U}}_{100}\right)$ as the estimate of *B*_0_/*U*_0_, where ${\bar{B}}_{100}$ and ${\bar{U}}_{100}$ were the mean of the 30 replicates of *B*_100_ and *U*_100_, respectively

*m*	SIZE20, MI-2, Anal-2			
	Control ${\hat{\gamma }}_{0}=\gamma_{m}$	Improved		
		${\hat{\gamma }}_{0}=\gamma_{m\geq 100}$	${\hat{\gamma }}_{0}=c_{m}/\left(1+c_{m}\right)$	${\hat{\gamma }}_{0}=\gamma_{m}\left(m/\left(1+m\right)\right)$
3	0.00379		0.00283	0.00284
5	0.00318		0.00265	0.00265
10	0.00286		0.00260	0.00260
20	0.00293		0.00279	0.00279
30	0.00278		0.00269	0.00269
40	0.00273		0.00267	0.00267
60	0.00277		0.00273	0.00273
80	0.00272		0.00269	0.00269
100	0.00270	0.00270	0.00267	0.00267
(∞)	Best ${\hat{\gamma }}_{0}$: 0.00267			
